# Unlocking the potential of biofilm-forming plant growth-promoting rhizobacteria for growth and yield enhancement in wheat (*Triticum* *aestivum* L.)

**DOI:** 10.1038/s41598-024-66562-4

**Published:** 2024-07-05

**Authors:** Munazza Rafique, Muhammad Naveed, Muhammad Zahid Mumtaz, Abid Niaz, Saud Alamri, Manzer H. Siddiqui, Muhammad Qandeel Waheed, Zulfiqar Ali, Abdul Naman, Sajid ur Rehman, Martin Brtnicky, Adnan Mustafa

**Affiliations:** 1 Soil Bacteriology Section, Agricultural Biotechnology Research Institute, AARI, Faisalabad, 38000 Pakistan; 2 Institute of Soil and Environmental Sciences, University of Agriculture, Faisalabad, 38040 Pakistan; 3 College of Agronomy, Gansu Agricultural University, Lanzhou 730070, China, Lahore, Pakistan; 4 Department of Botany and Microbiology, College of Science, King Saud University, Riyadh, 11451 Saudi Arabia; 5 Wheat Breeding Group, Plant Breeding and Genetics Division, Nuclear Institute for Agriculture and Biology (NIAB), Faisalabad, 38000 Pakistan; 6 Department of Plant Breeding and Genetics, University of Agriculture, Faisalabad, 38040 Pakistan; 7 Department of Chemistry, University of Agriculture, Faisalabad, 38040 Pakistan; 8 Agricultural Biotechnology Research Institute, AARI, Faisalabad, 38000 Pakistan; 9 Department of Agrochemistry, Soil Science, Microbiology and Plant Nutrition, Faculty of AgriSciences, Mendel University in Brno, Zemedelska 1, 61300 Brno, Czech Republic; 10 Key Laboratory of Vegetation Restoration and Management of Degraded Ecosystems, South China Botanical Garden, Chinese Academy of Sciences, Guangzhou, 510650 China; 11 Director, Programs and Projects Department, Islamic Organization for Food Security, 019900 Astana, Kazakhstan

**Keywords:** Plant sciences, Environmental sciences

## Abstract

Plant growth-promoting rhizobacteria (PGPR) boost crop yields and reduce environmental pressures through biofilm formation in natural climates. Recently, biofilm-based root colonization by these microorganisms has emerged as a promising strategy for agricultural enhancement. The current work aims to characterize biofilm-forming rhizobacteria for wheat growth and yield enhancement. For this, native rhizobacteria were isolated from the wheat rhizosphere and ten isolates were characterized for plant growth promoting traits and biofilm production under axenic conditions. Among these ten isolates, five were identified as potential biofilm-producing PGPR based on in vitro assays for plant growth-promoting traits. These were further evaluated under controlled and field conditions for their impact on wheat growth and yield attributes. Surface-enhanced Raman spectroscopy analysis further indicated that the biochemical composition of the biofilm produced by the selected bacterial strains includes proteins, carbohydrates, lipids, amino acids, and nucleic acids (DNA/RNA). Inoculated plants in growth chamber resulted in larger roots, shoots, and increase in fresh biomass than controls. Similarly, significant increases in plant height (13.3, 16.7%), grain yield (29.6, 17.5%), number of tillers (18.7, 34.8%), nitrogen content (58.8, 48.1%), and phosphorus content (63.0, 51.0%) in grains were observed in both pot and field trials, respectively. The two most promising biofilm-producing isolates were identified through 16 s rRNA partial gene sequencing as *Brucella* sp. (BF10), *Lysinibacillus macroides* (BF15). Moreover, leaf pigmentation and relative water contents were significantly increased in all treated plants. Taken together, our results revealed that biofilm forming PGPR can boost crop productivity by enhancing growth and physiological responses and thus aid in sustainable agriculture.

## Introduction

Over the past three decades, chemical fertilization in intensive agriculture has become a public concern due to its negative impacts on the environment and human health. Therefore, new strategies are needed to boost agricultural productivity for sustainable and friendly environment ^1,2^. Among various strategies, the application of beneficial microbes stands out as a crucial solution for addressing crop productivity issues in agriculture. Soil microorganisms, specifically plant growth promoting rhizobacteria (PGPR) are crucial for agriculture because they increase soil nutrient availability and promote plant health^3^. However, their potential for nutrient availability and plant defense mechanisms has not yet been fully understood ^4^.

PGPR usually enhance plant growth because they colonize plant roots and protect them from harmful microbes^5^. The specific rhizobacteria required for adhesion and subsequent colonization play a critical role in successful bacterial root colonization ^6,7^. In addition, several biotic and abiotic factors, including temperature, water content, pH, the composition of root exudates, mineral concentrations, and abundance of microbes have an enormous effect on how rhizobacteria interact with plant roots and colonize them^8^. PGPR can inhabit the root zone as a well-organized bacterial community adhered to a biotic or abiotic surface called biofilm. Such PGPR can also establish biofilm-like structures composed of multi-bacterial communities in the rhizosphere. They are considered responsible for this phenomenon as a survival strategy and protect the plant under adverse conditions ^9^. The roots associated microbial biofilms can increase soil fertility and crop production. Using biofilm-associated rhizobacteria offers significant advantages for the bio-organic market sector. Researchers are investigating the potential of biofilm-forming rhizobacteria as an alternative inoculum to address nutrient deficiencies and enhance crop yield production.

Biofilm is a structured community of dense microbial colonies of one or more microbial species clinging to an abiotic or biotic surface and enveloped in an extracellular matrix (ECM) ^10^. It has high cell density ranging from 10^8^ to 10^11^ cells g^−1^ wet weight basis ^11^. The bacteria can form a single- or multi-layered biofilm on the surface, with single or many bacterial species within the ECM^12^. The ECM of biofilm provides mechanical stability, promotes cell-cell communication, and induces synergistic micro-consortia that distinguish the biofilm lifestyle from the planktonic state ^13^. Interestingly, microbial cells are found as biofilms rather than their planktonic counterparts in natural habitats. This suggests that the biofilm-forming rhizobacterial approach may be a more viable and long-lasting alternative than conventional bacterial inoculums^14^. Despite of their huge importance, biofilm-forming rhizobacterial inoculants are less explored than traditional rhizobacterial strains and other bacterial community structures. They are found in various niches within agroecosystems ^15^. Application of biofilm-forming rhizobacteria may improve bacterial cell survival during their initial introduction into natural soils and flourish over time. Diverse rhizobacteria can develop microcolonies on various root zones, ranging from the root tip to the elongation zone, and produce abundant populations and mature biofilms ^16^.

Research on root colonization patterns shows that rhizobacteria form microcolonies or aggregates on root surfaces within the rhizosphere, albeit in an uneven and non-uniform manner^17–19^. This has spurred our investigation into the use of biofilm-forming rhizobacteria for enhanced crop production. Prior studies have tested biofilm-forming rhizobacterial inoculants for crop yield improvement mainly in pot trials, with few extending to field trials^20^. Consequently, our study aims to (i) identify and characterize novel biofilm-forming rhizobacteria from the wheat rhizosphere for their in vitro plant growth-promoting traits, and (ii) evaluate their effectiveness in boosting wheat growth and yield in both controlled and field settings.

## Results

### Isolation of biofilm-forming rhizobacteria

**Figure 1:**
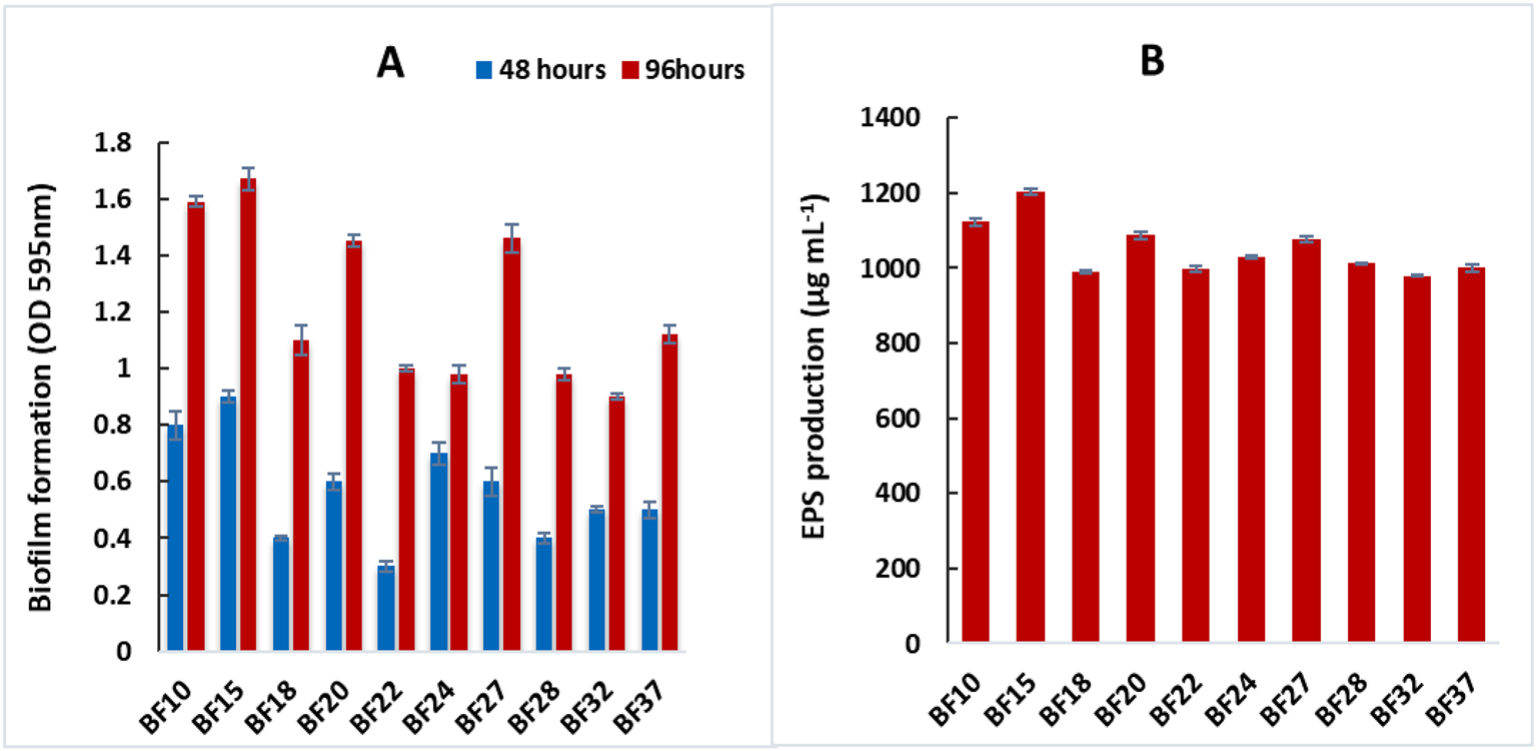
In vitro screening of bacterial strains for biofilm formation **(A)** and exopolysaccharide production **(B)**. The error bars represent the least significant difference among treatments at *P* ≤ 0.05.

Over fifty rhizobacterial isolates from the wheat rhizosphere were screened for biofilm formation. Ten isolates (BF10, BF15, BF18, BF20, BF22, BF24, BF27, BF28, BF32, BF37) were confirmed as biofilm-forming strains (Fig. 1A). These strains exhibited optical density readings (OD_595_) ranging from 0.31 to 0.92 after 48 hours of incubation, and from 0.92 to 1.67 after 96 hours, indicating their growth and proliferation over time. Strain BF15 displayed the highest biofilm formation, reaching an OD_595_ of 0.92 after 48 hours and 1.67 after 96 hours of incubation. Following closely behind, strain BF10 exhibited substantial biofilm formation with an OD_595_ of 0.80 and 1.59 after 48 and 96 hours, respectively. The exopolysaccharides (EPS) production by tested strains ranged from 978.0 to 1203.0 µg mL^−1^ (Fig. 1B). The maximum EPS production was reported by strain BF15 (1203.0 µg mL^−1^) followed by strain BF10 (1122.0 µg mL^−1^). The minimum EPS production of 978.0 µg mL^−1^ was reported by strain BF32.

### Characteristics and identification of biofilm-forming rhizobacteria

The gram staining revealed that, all the tested biofilm-forming rhizobacterial strains were gram-positive except BF10 and exhibited a characteristic rod shape (Table 1). All strains were aerobic and motile with colony sizes of large for (BF28), medium for (BF10, BF15, BF24, BF27, BF37), and small for (BF18, BF20, BF22, BF32), as described in (Table 1). All isolates tested positive for catalase, glucose utilization, starch hydrolysis, and oxidase tests. However, isolate BF37 tested negative for glucose utilization.

**Table 1:** Morphological, biochemical and PGP characteristics of biofilm-forming bacterial strains.

Strains	Gram reaction	Cell shape	Colony shape and size	Motility	Nature	Catalase test	Oxidase test	Glucose utilization	Starch hydrolysis	IAA (µg mL^−1^)	SP assay	PS activity	KS activity	ST (%)
BF10	−ve	Rod	Round medium	Motile	Aerobic	+	+	+	+	13.2 ± 1.12*	+	+	+	2.5
BF15	+ ve	Rod	Round medium	Motile	Aerobic	+	+	+	+	15.4 ± 1.02	+	+	+	2.5
BF18	+ ve	Rod	Round small	Motile	Aerobic	+	+	+	+	10.2 ± 1.10	−	+	−	1.5
BF20	+ ve	Rod	Round small	Motile	Aerobic	+	+	+	+	9.1 ± 1.00	+	+	−	2.0
BF22	+ ve	Rod	Round small	Motile	Aerobic	+	+	+	+	8.6 ± 1.45	−	−	+	1.5
BF24	+ ve	Rod	Round medium	Motile	Aerobic	+	+	+	+	10.9 ± 1.18	+	+	+	2.5
BF27	+ ve	Rod	Round medium	Motile	Aerobic	+	+	+	+	11.9 ± 1.92	+	+	+	2.0
BF28	+ ve	Rod	Round Large	Motile	Aerobic	+	+	+	+	7.6 ± 0.96	+	+	−	1.5
BF32	+ ve	Rod	Round small	Motile	Aerobic	+	+	+	+	9.9 ± 0.99	+	−	+	1.0
BF37	+ ve	Rod	Wavy medium	Motile	Aerobic	+	+	−	+	6.8 ± 0.78	+	−	−	1.5

*The data is average of three replicates ± SE. The symbol, + ve means Gram-positive, −ve means Gram-negative, − means absence of the trait, and + ve means presence of the trait. IAA: Indole-3-acetic acid, SP: Siderophore production, PS: Phosphorus solubilization, KS: potassium solubilization ST: Salt tolerance.

Biofilm-forming rhizobacterial strains (BF10, BF15, BF18, BF20, BF22, BF24, BF27, BF28, BF32, and BF37) were screened for plant growth promoting (PGP) traits, including IAA, siderophores, solubilization of phosphate and potassium and salt stress tolerance. They produced IAA in the presence of L-tryptophan, ranging from 6.8 to 15.4 µg mL^−1^ (Table 1). The maximum IAA production (15.4 µg mL^−1^) was obtained from strain BF15, followed by strain BF10 which reported 13.2 µg mL^−1^. The minimum IAA production of 6.8 µg mL^−1^ was reported by strain BF37. All the tested strains were positive for siderophore production except BF18 and BF22. The tested strains also showed solubilization of insoluble phosphate on the Pikovskya agar medium except for strains BF22, BF32, and BF37. Sixty percent of the biofilm-forming rhizobacterial strains were also positive for the solubilization of potassium.

Moreover, the salt tolerance ability of all strains was assessed by adding salt with a range of 1.5–2.5% in tryptic soya broth. All isolates showed different capabilities to tolerate salt concentration. But BF10, BF15, and BF24 showed maximum tolerance against a salt concentration of 2.5%.

**Figure 2:**
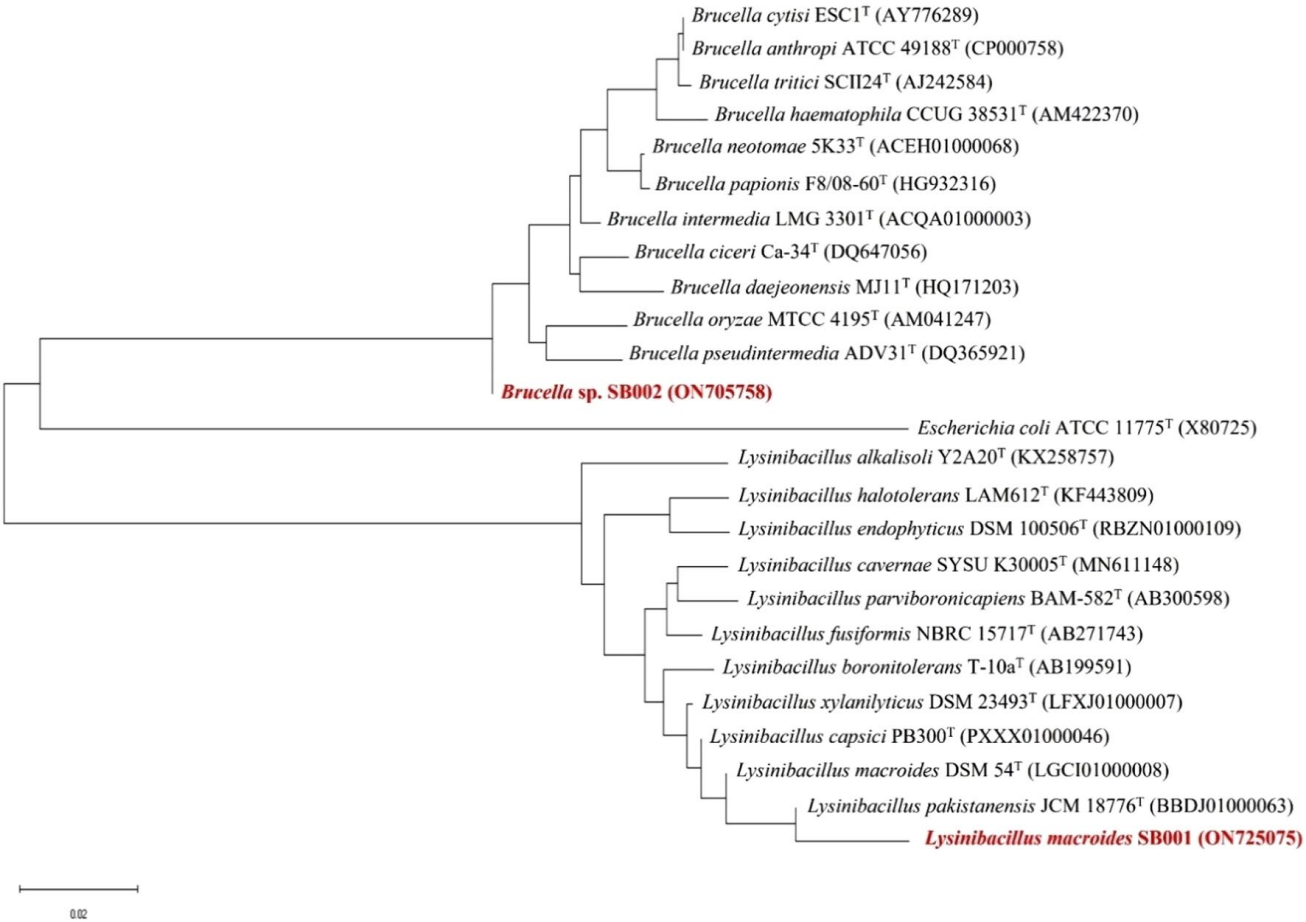
Phylogenetic tree of isolated bacterial strains BF10 and BF15 (SB002 and SB001 respectively) based on 16S rRNA gene sequences constructed using the neighbor-joining method.

Two promising biofilm-producing isolates were identified through partial 16S rRNA gene sequencing: isolate BF10 as *Ochrobactrum intermedium* sp. (Gene Bank accession number ON705758.1) and isolate BF15 as *Lysinibacillus macroides* (Gene Bank accession number ON725075.1). All the other bacterial isolates were identified on the basis of morphological and biochemical features as *Bacillus* sp. conferring to Bergey's manual of determinative bacteriology. The phylogenetic tree of 16S rRNA gene of these bacterial isolates also showed that they belong to the genera *Lysinibacillus* and *Brucella* (Fig. 2).

### Mean surface enhanced Raman spectroscopy (SERS) spectra

**Figure 3:**
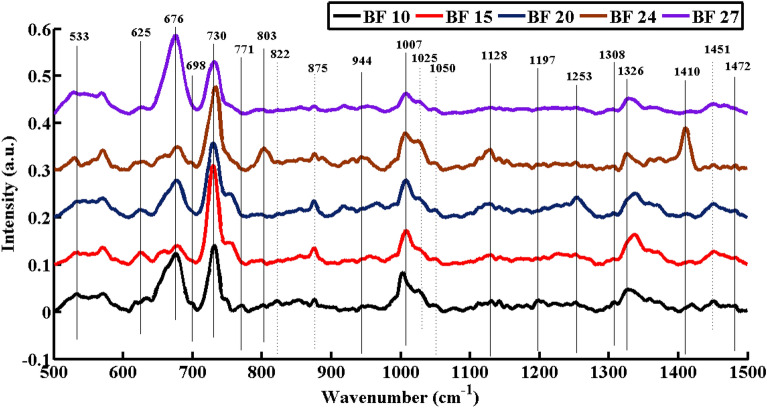
Mean SERS spectra of selected biofilm-forming bacterial strains.

The SERS peak analysis describe the relative efficacy of different strains to produce various components of biofilm. The analysis showed some SERS spectral characteristics in SERS mean plot which clearly differentiate the biochemical features of selected biofilm-forming bacterial strains BF10, BF15, BF20, BF24 and BF27 (Fig. 3). Most of the biochemical contents of biofilm were associated with proteins, carbohydrates, lipids, amino acids and DNA/RNA (Table 2). The differentiating SERS bands are labelled by solid lines while SERS bands with intensity-based differences are denoted with dotted lines.

**Table 2:** SERS peak assignment of different biofilm- forming bacterial strains.

SERS peaks (cm^−1^)	Peak assignment	Components	References
533	S–S str., COC glycosidic ring def	Carbohydrates	^108^
625	d(NCO) Tyrosine	DNA/RNA	^109^
676	Guanine	DNA/RNA	^108^
698	-C-S- stretching vibrations	Protein	^110^
730	Adenine	DNA/RNA	^111^
771	polysaccharides; COO − wagging and C–C skeletal	Carbohydrates	^112^
803	n(PO_2_), n(CC) ring breathing	DNA	^113^
822	Tyrosine	DNA/RNA	^114^
875	CC stretching, COC 1,4-glycosidic linkage saccharides	Proteins	^115^
944	Lipid components	Lipids	^116^
1007	CC aromatic ring –phenylalanine, and skeletal stretching of tryptophan; CCH stretching of carotenoids	Proteins	^43,117^
1025	n(CC) aromatic ring, –phenylalanine, and skeletal stretching of tryptophan	Proteins	^118^
1050	Carbohydrates, C–C, C–O, –C–OH def	Carbohydrates	^119^
1128	CC unsaturated fatty acids	Lipids	^120^
1197	Tyrosine	DNA/RNA	^121^
1252–1260	Amide III	Proteins	^108^
1326	Tyrosine Ring vibration /CH vibration	Proteins	^122–124^
1410	ν (CO2) (α -amino acids)	Proteins	^44,125^
1451	CH_2_ scissoring (phospholipids and fatty acid)	Proteins	^126^
1472	CH_2_ deformation (lipids)	Lipids	^127^

### Evaluation for in vitro plant growth promotion

Biofilm-forming rhizobacterial strains (BF10, BF15, BF18, BF20, BF22, BF24, BF27, BF28, BF32, and BF37) significantly increased the wheat germination and seedling growth compared to the control (Table 3).

**Table 3:** Effect of biofilm- forming bacterial isolates on growth promotion of wheat seedlings under axenic conditions.

Biofilm-forming strains	Seedling germination (%)	Shoot length (cm)	Root length (cm)	Shoot fresh weight (g plant^−1^)	Root fresh weight (g plant^−1^)
Control	79.0 ± 1.2*	7.7 ± 0.02	8.1 ± 0.13	0.60 ± 0.0002	0.36 ± 0.0004
BF10	90.0 ± 1.0	9.5 ± 0.05	9.2 ± 0.02	0.80 ± 0.0001	0.50 ± 0.0010
BF15	92.0 ± 1.4	10.9 ± 0.04	9.5 ± 0.14	0.88 ± 0.0005	0.52 ± 0.0002
BF18	85.0 ± 1.1	8.0 ± 0.08	8.4 ± 0.11	0.72 ± 0.0011	0.42 ± 0.0011
BF20	91.0 ± 0.8	8.5 ± 0.06	9.0 ± 0.05	0.80 ± 0.0004	0.48 ± 0.0003
BF22	82.0 ± 1.8	8.8 ± 0.01	8.9 ± 0.03	0.77 ± 0.0022	0.44 ± 0.0012
BF24	86.0 ± 0.7	9.5 ± 0.07	9.0 ± 0.04	0.74 ± 0.0007	0.45 ± 0.0020
BF27	89.0 ± 0.6	8.7 ± 0.09	9.2 ± 0.06	0.76 ± 0.0031	0.44 ± 0.0019
BF28	83.0 ± 0.9	8.1 ± 0.10	8.8 ± 0.01	0.70 ± 0.0009	0.41 ± 0.0014
BF32	84.0 ± 1.5	8.4 ± 0.03	8.3 ± 0.08	0.68 ± 0.0012	0.39 ± 0.0013
BF37	84.0 ± 0.4	8.2 ± 0.12	8.5 ± 0.05	0.69 ± 0.0006	0.38 ± 0.0009

The data is average of three replicates ± SE.

The lowest seed germination was recorded in the control group, but it improved with the application of biofilm-producing rhizobacterial strains. Strain BF15 increased germination by up to 16.5%, followed by BF20 with a 15.2% increase. Similarly, shoot and root lengths were highest with strain BF15, showing increases of 41.6% and 17.3%, respectively, compared to the control. Data regarding shoot and root fresh weight depicted a significant response to seed inoculation with biofilm-forming rhizobacterial strains. The highest increase in shoot and root fresh weight of 46.7 and 44.4%, respectively, was observed with the application of BF15. The strains BF10 also showed a better increase of 33.3 and 38.9% in shoot and root fresh weight, respectively.

### Promotion of wheat growth and yield in pot experiment

From a pool of ten biofilm-forming rhizobacterial strains, the top five performers, BF10, BF15, BF20, BF24, and BF27,exhibited notable enhancements in wheat growth and yield characteristics during the pot trial (Table 4). Strain BF15 showed the highest shoot length (13.3% increase) and BF10 (9.6% increase) over control. Both strains were statistically non-significant compared to other tested strains except for strain BF20 which was statistically similar to control. The highest increase of 20.0% in shoot fresh mass was reported by strain BF15 which was statistically at par with strain BF27. The strains BF10, BF15, and BF20 reported higher shoot dry mass with increase of 18.42, 23.68 and 18.42%, respectively as compared to control. Although these strains did not show significant differences in shoot dry mass among themselves, they were statistically significant compared to the control group. Strain BF15 reported maximum root length, root fresh mass and root dry mass, with increases of 23.93, 30.30 and 22.27% respectively over control (Table 4). The maximum tiller count plant^−1^, straw weight, grain weight, and total yield were reported by strain BF15 with increases of 18.72, 35.52 and 32.65%, respectively, compared to control. The highest increases of 46.71, 40.79, and 31.58% in 1000 grain weight was reported by strains BF15, BF27, and BF20, respectively, compared to control.

**Table 4:** Effect of biofilm-forming bacterial strains on the growth characteristics of wheat under wire house experiment.

Treatments	Control	BF10	BF15	BF20	BF24	BF27	LSD
Shoot length (cm)	76.80 ± 1.09 c	84.20 ± 1.32 ab	87.00 ± 0.98 a	81.00 ± 0.87 bc	82.00 ± 0.76 ab	82.00 ± 0.93 ab	5.008
Shoot fresh mass (g plant^-1^)	15.63 ± 0.31 d	17.83 ± 0.09 b	18.75 ± 0.03 a	17.95 ± 0.98 b	17.63 ± 0.67 c	18.0 ± 0.21 ab	4.962
Shoot dry mass (g plant^−1^)	3.80 ± 0.029 c	4.50 ± 0.03 ab	4.70 ± 0.04 a	4.50 ± 0.05 ab	4.30 ± 0.01 b	4.40 ± 0.03 b	0.682
Root length (cm)	48.01 ± 1.08 c	55.03 ± 2.09 ab	59.50 ± 0.87 a	53.50 ± 1.02 bc	54.02 ± 0.90 ab	52.50 ± 0.89 bc	5.597
Root fresh mass (g plant^−1^)	16.50 ± 0.20 d	19.50 ± 0.98 b	21.50 ± 0.87 a	18.30 ± 0.09 c	18.80 ± 0.30 bc	18.70 ± 0.76 bc	3.009
Root dry mass (g plant^−1^)	4.40 ± 0.019 c	4.81 ± 0.03 ab	5.38 ± 0.02 a	4.50 ± 0.01 b	4.69 ± 0.01 b	4.70 ± 0.02 b	1.180
Tillers count plant^−1^	4.06 ± 0.03 d	4.64 ± 0.09 b	4.82 ± 0.07 a	4.63 ± 0.01 b	4.38 ± 0.05 c	4.38 ± 0.02 c	0.520
Straw weight (g plant^−1^)	7.60 ± 0.43 d	9.80 ± 0.76 ab	10.30 ± 0.87 a	9.17 ± 0.76 c	9.55 ± 0.56 b	9.35 ± 0.49 b	0.709
Grain weight (g plant^−1^)	7.10 ± 0.21 d	8.60 ± 0.45 b	9.20 ± 0.98 a	7.90 ± 0.12 c	8.10 ± 0.01 bc	7.60 ± 0.11 c	0.617
1000 grain weight (g)	30.40 ± 1.23c	39.80 ± 1.12 ab	44.60 ± 1.11 a	40.00 ± 0.99 ab	32.20 ± 1.10 b	42.80 ± 1.24 ab	3.868
Total yield (g plant^−1^)	14.70 ± 0.45 e	18.40 ± 0.21 b	19.50 ± 0.34 a	17.07 ± 0.22 d	17.65 ± 0.31 c	16.95 ± 0.56 d	1.118
Grain: straw ratio	0.93 ± 0.03 a	0.88 ± 0.06 b	0.89 ± 0.10 b	0.86 ± 0.02 c	0.85 ± 0.09 c	0.81 ± 0.05 d	0.066
Grain N (%)	2.04 ± 0.05 c	2.57 ± 0.01 b	3.24 ± 0.02 a	2.73 ± 0.06 b	3.18 ± 0.04 a	2.80 ± 0.07 b	0.317
Straw N (%)	1.04 ± 0.03 e	1.42 ± 0.07 d	2.02 ± 0.05 a	1.50 ± 0.06 cd	1.86 ± 0.001 b	1.54 ± 0.002 c	0.113
Grain P (%)	1.11 ± 0.002 f.	1.70 ± 0.051 b	1.81 ± 0.028 a	1.54 ± 0.010 c	1.50 ± 0.004 d	1.40 ± 0.003 e	0.031
Straw P (%)	0.67 ± 0.003 d	0.86 ± 0.006 b	1.02 ± 0.001 a	0.77 ± 0.009 c	0.75 ± 0.002 cd	0.70 ± 0.004 cd	0.085

Mean standard deviation, n = 3; values followed by the same letter in a column were not significantly different (p ≤ 0.05).

The control treatment reported a maximum grain-to-straw ratio followed by strains BF15 and BF10. The nutrient concentration was significantly promoted through the application of biofilm-forming rhizobacterial strains. The highest N contents were found in grain and straw with increases of 58.8 and 94.2%, respectively, over control with the application of strain BF15 (Table 4). Interestingly, inoculation with the same strain also showed maximum P contents in grain and straw with increment of 63.1 and 52.2%, respectively, over control (Table 4).

### Promotion of wheat physiology and crop productivity under field conditions

**Figure 4:**
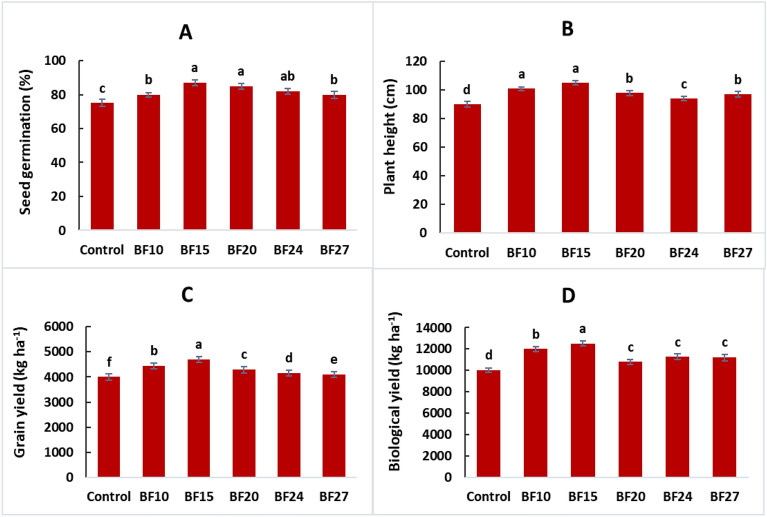
Effect of biofilm-forming bacterial strains on wheat seed germination **(A)**, plant height **(B),** grain yield **(C)**, and biological yield **(D)** under field conditions. The error bars represent the least significant difference among treatments at *P* ≤ 0.05.

**Figure 5:**
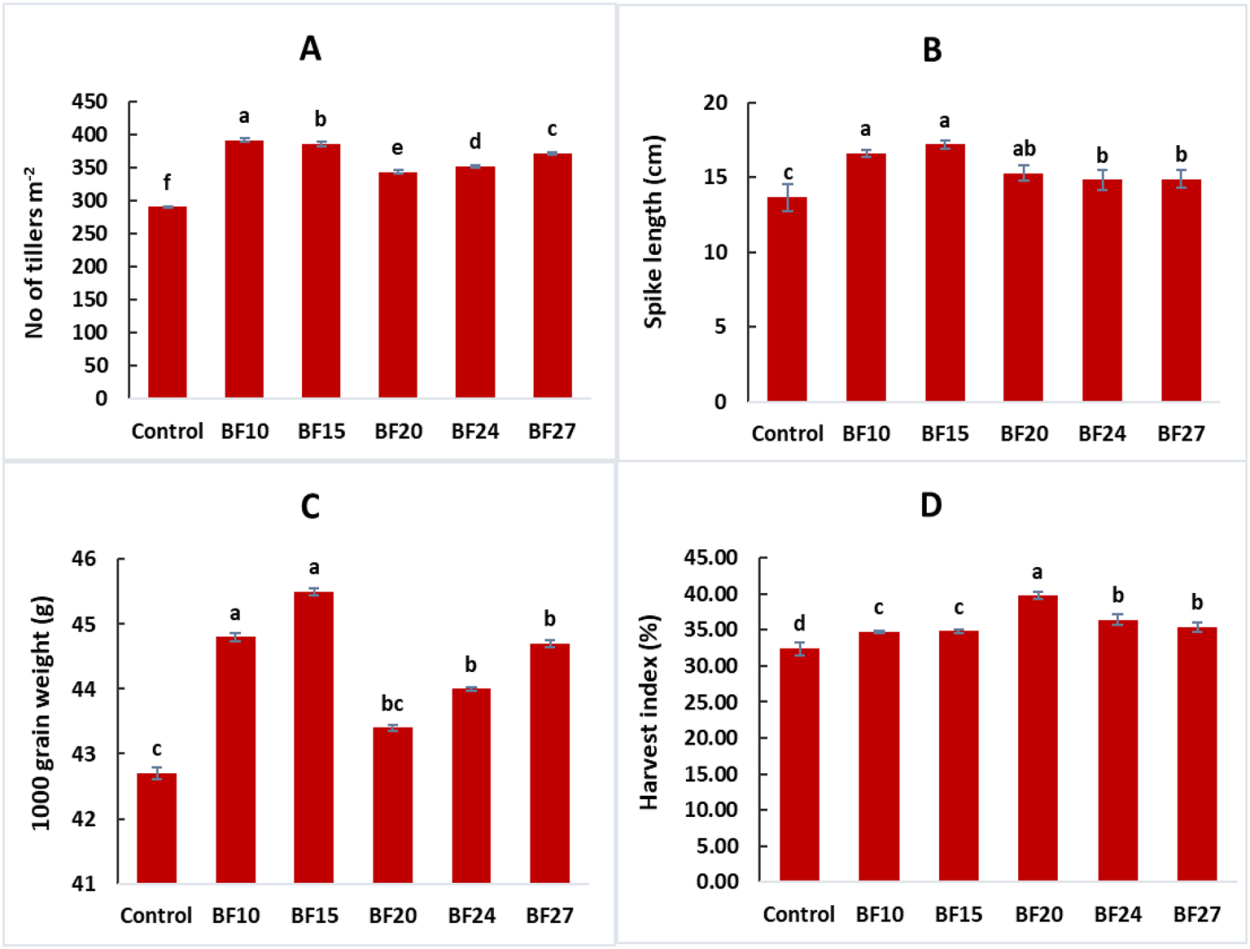
Effect of biofilm-forming bacterial strains on number of tillers **(A)**, spike length **(B)**, 1000 grain weight **(C)** and harvest index **(D)** of wheat under field conditions. The error bars represent the least significant difference among treatments at *P* ≤ 0.05.

Over two years (2020–2021, 2021–2022), wheat growth and yield attributes significantly improved with the application of five selected efficient strains (BF10, BF15, BF20, BF24, and BF27) compared to the uninoculated control All the tested strains improved the seed germination of wheat, but a maximum increase in germination of 16.0% was observed in strain BF15 treated plants, compared to control (Fig. 4A). This treatment was statistically similar to strains BF20 and BF24, however, these treatments were significantly different from control. Strain BF15 reported a maximum increase of 16.7% in plant height over control (Fig. 4B). Strain BF20 also reported a better increase in plant height by 8.9% compared to control. The maximum grain and biological yield were reported by BF15 (17.5 and 15.0%, respectively increase over control) followed by isolate BF10 (Fig. 4C, D). The maximum increase in the number of tillers m^−2^ was up to 34.8% over control by inoculation with BF10 followed by BF15 which showed an increase in the number of tillers up to 32.8% compared to control (Fig. 5A). The strains BF10 and BF15 significantly promoted spike length and 1000 grain weight up to 25.5 and 6.6%, respectively, over uninoculated control (Fig. 5 B,C). Inoculation with strain BF20 significantly promoted the harvest index with an increase of up to 22.6% compared to control (Fig. 5D).

**Figure 6:**
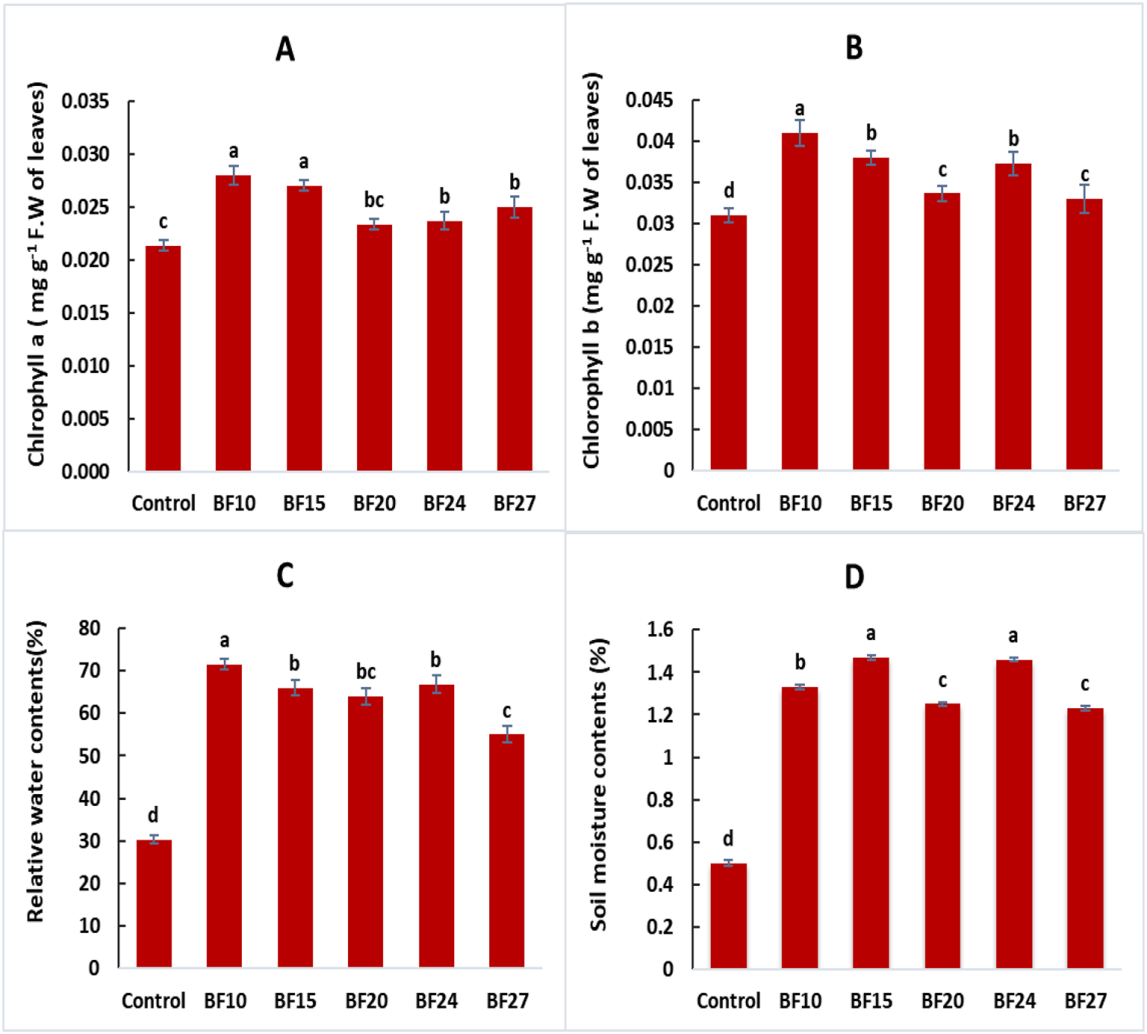
Effect of biofilm-forming bacterial strains on wheat leaves chlorophyll a contents **(A)** chlorophyll b contents **(B)** relative water contents **(C)** and soil moisture contents **(D)** under field conditions. The error bars represent the least significant difference among treatments at *P* ≤ 0.05.

**Figure 7:**
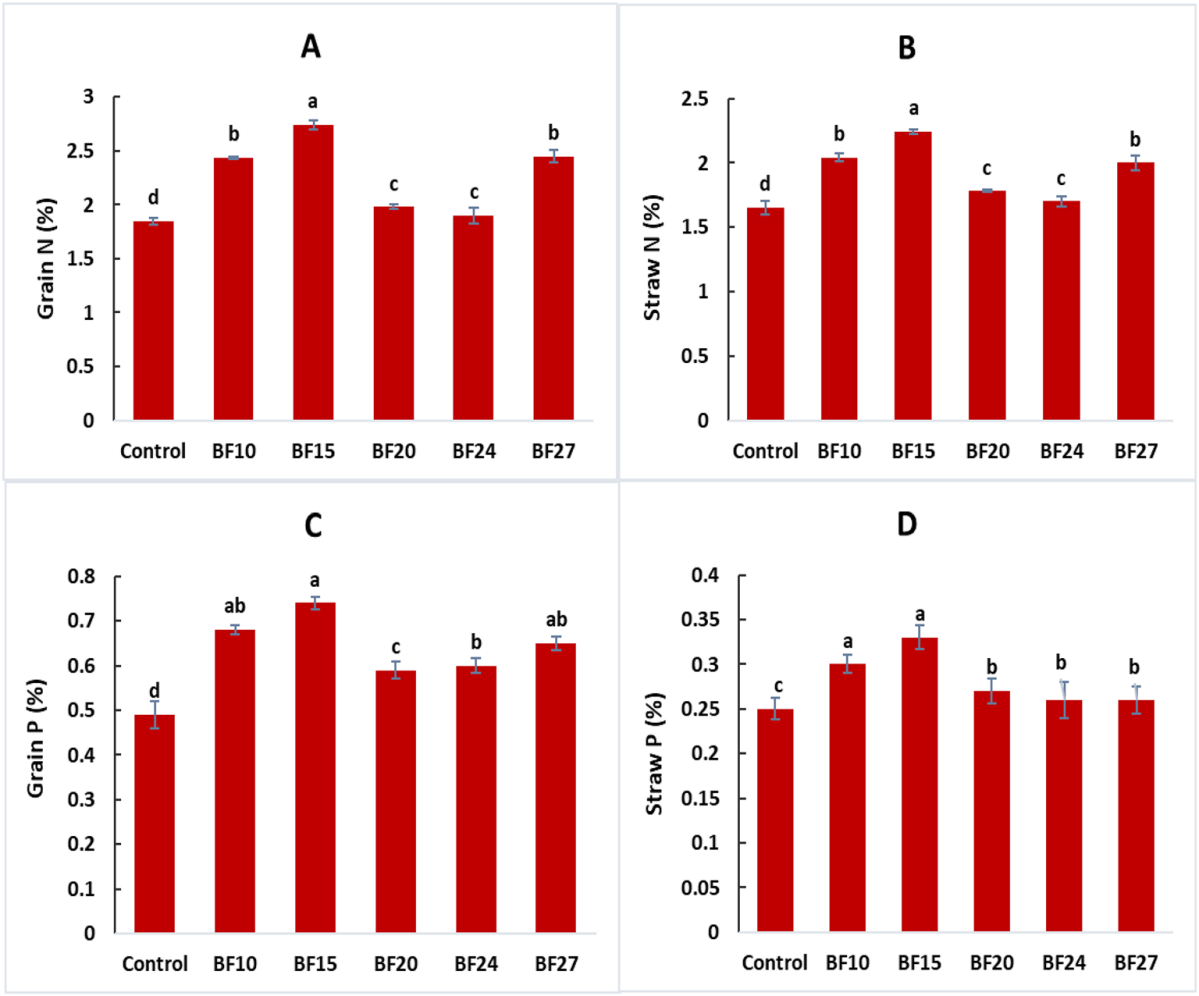
Effect of biofilm-forming bacterial strains grain N **(A)**, straw N **(B),** grain P **(C)** and straw P contents **(D)** in wheat under field conditions. The error bars represent the least significant difference among treatments at *P* ≤ 0.05.

The strains BF10 and BF15 reported the maximum increase in chlorophyll a content with increase of 33.3 and 28.6%, respectively, over control. The chlorophyll b contents were highest by inoculation with BF10 (32.3% increase) followed by isolates BF15 and BF24 over control (Fig. 6 A,B). The enhancement in relative water contents and soil moisture contents were observed by inoculating wheat seeds with biofilm-forming rhizobacterial strains (Fig. 6C, D). The highest increase in RWC by 135.8% was observed after inoculation with BF10 followed by BF15 which reported 117.7% higher RWC, compared to control. The highest increase in soil moisture contents was observed by inoculation with strains BF15 and BF24 with increase of 194.0 and 192.0%, respectively, over uninoculated controlUnder field conditions, inoculating biofilm-forming rhizobacterial strains significantly influenced the nutrient contents of wheat crops (Fig. 7). The highest increase in nitrogen content in grains (48.1%) and straw (35.8%) was observed with inoculation of strain BF15 compared to the control (Fig. 7A, B). The maximum P contents in grains with an increase of 51.0% were also found by inoculation with BF15, followed by BF10 and BF27 which caused 38.8 and 32.7%, respectively, more P contents in grains over control. The straw P concentration was highest by inoculation with BF15 and BF10 having 32.0 and 20.0%, respectively, more P contents in straw, compared to control (Fig. 7C, D).

### Correlation matrix and principal component analysis

**Figure 8:**
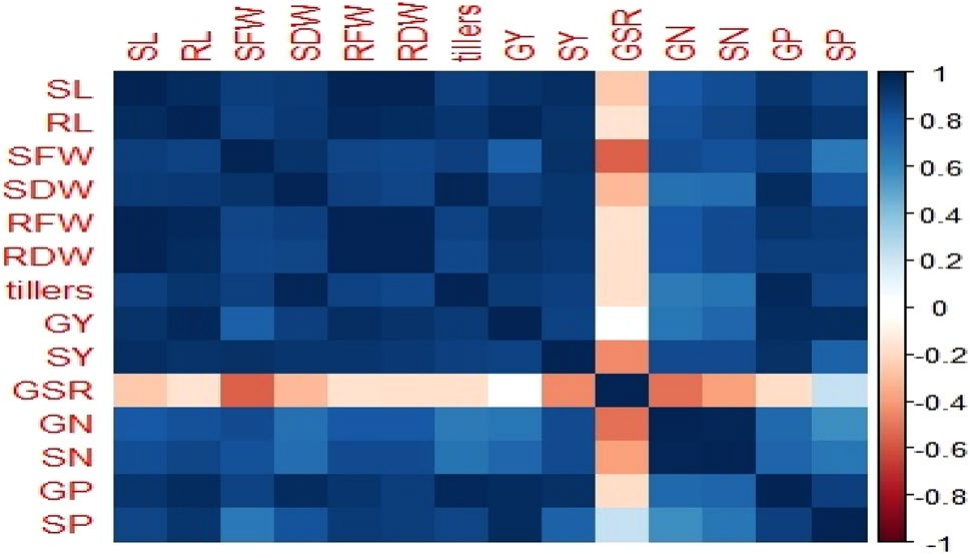
Correlation among measured parameters of wheat grown in pot trial. The abbreviations of the correlation matrix are as shoot length (SL), root length (RL), shoot fresh weight (SFW), shoot dry weight (SDW), root fresh weight (RFW), root dry weight (RDW), number of tillers (tillers), grain yield (GY), straw yield (SY), grain: straw ratio (GSR), grain N (GN), straw N (SN), grain P (GP), and straw P (SP). Positive correlations are displayed in blue and negative correlations in red color. The color intensity and the size of the circle are proportional to the correlation coefficients.

**Figure 9:**
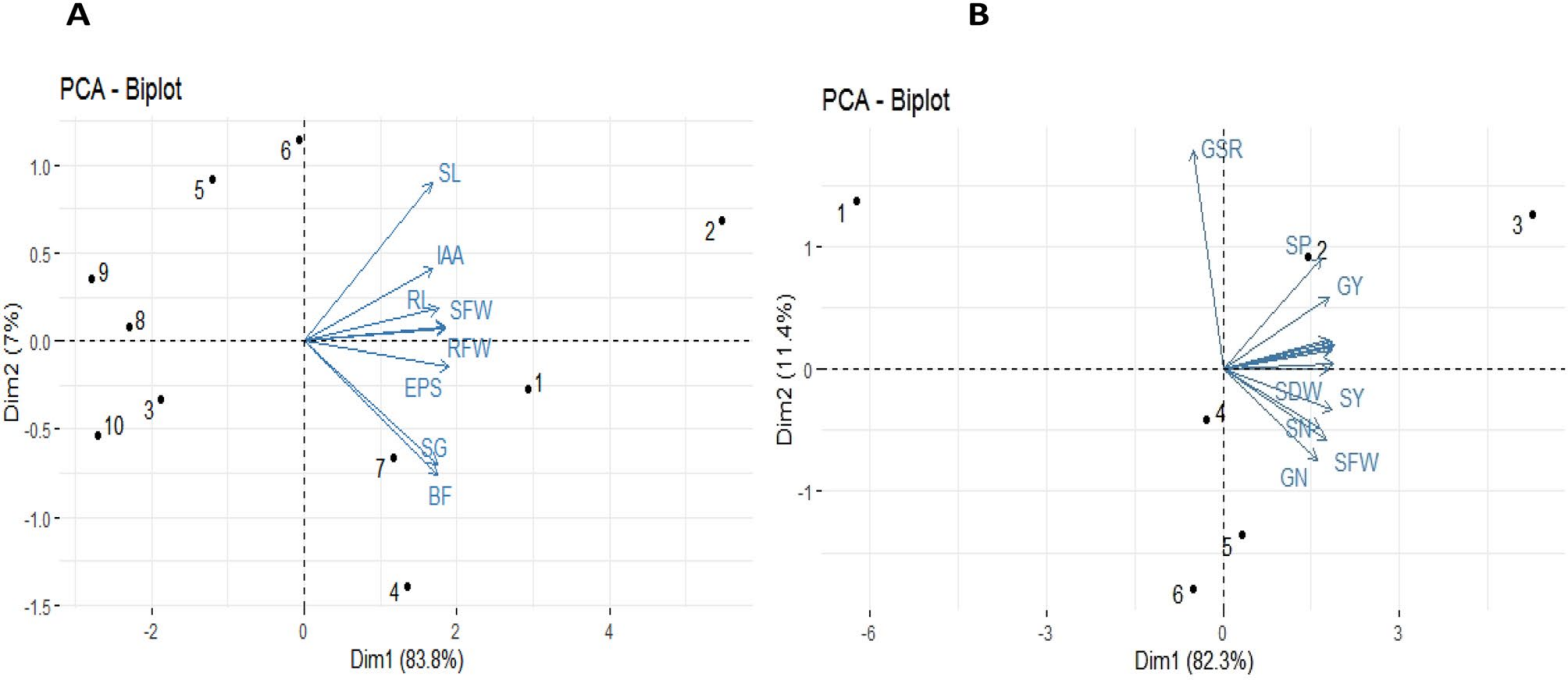
Represents the PCA biplot among measured parameters of biofilm producing PGPR under lab conditions. Treatments are as T_1_ (BF10), T_2_ (BF15), T_3_ (BF18), T_4_ (BF20), T_5_ (BF22), T_6_ (BF24), T_7_ (BF27), T_8_ (BF28), T_9_ (BF32), T_10_ (BF37) and abbreviations of biplot are as Shoot length (SL), Root length (RL), Shoot fresh weight (SFW), Root fresh weight (RFW), Exopolysaccharide production (EPS), Indole acetic acid production (IAA), Seed germination (SG), Biofilm formation (BF) **(A).** Represents the PCA biplot among measured parameters of wheat grown in pot experiment. Treatments are as T_1_ (Control), T_2_ (BF10), T_3_ (BF15), T_4_ (BF20), T_5_ (BF24), T_6_ (BF27) and the abbreviations of the biplot are as shoot length (SL), root length (RL), shoot fresh weight (SFW), shoot dry weight (SDW), root fresh weight (RFW), root dry weight (RDW), number of tillers (tillers), grain yield (GY), straw yield (SY), grain: straw ratio (GSR), grain N (GN), straw N (SN), grain P (GP), straw P (SP) **(B)**.

A strong positive correlation was observed in all studied growth and yield attributes and mineral contents of wheat in the pot study (Fig. 8). The first biplot (Fig. 9) showed that among all the components, the first two components *viz*. PC1 (Dim1) and PC2 (Dim2) exhibited maximum contribution and accounted for 90.8% of the total dataset. Principal components 1 (Dim1) and 2 (Dim2) explained 83.8 and 7.0% of the variability among the variables studied, respectively (Fig. 9). The distribution of all treatments depicted that all isolates had positive effect on plant growth promoting traits. Treatments with isolates of biofilm-producing PGPR (BF10, BF15, BF 20, BF24, BF27) were displaced from isolates i.e., BF18, BF22, BF 28, BF32, BF37 and they have more pronounced effect. The first group of variables with which PC 1 is positively correlated includes: shoot length (SL), root length (RL), shoot fresh weight (SFW), root fresh weight (RFW), Exopolysaccharide production (EPS), indole acetic acid production (IAA), seed germination (SG), biofilm formation (BF).

The second biplot showed that among all the components, the first two components *viz*. PC1 (Dim1) and PC2 (Dim2) exhibited maximum contribution and accounted for 93.7% of the total dataset. Principal components 1 (Dim1) and 2 (Dim2) explained 82.3 and 11.4% of the variability among the variables studied, respectively. The distribution of all treatments depicted that all treated plants had a positive effect on all growth and yield parameters of the wheat crop over control. The PCA further confirmed that the treatment where isolate BF15 was used had a more pronounced effect in improving plant traits as compared to uninoculated control. The first group of variables with which PC 1 is positively correlated includes: shoot length (SL), root length (RL), shoot fresh weight (SFW), shoot dry weight (SDW), root fresh weight (RFW), root dry weight (RDW), number of tillers (tillers), grain yield (GY), straw yield (SY), grain to straw ratio (GSR), grain N (GN), straw N (SN), grain P (GP), and straw P (SP).

## Discussion

Bioinoculants are used in sustainable crop production to promote plant growth. The complicated process of plant-microbe interaction and environmental conditions determine how effectively bacterial inoculants promote crop production ^21,22^. The beneficial rhizosphere colonization of bacterial inoculants and the expression of PGP traits, including plant growth regulators, are important factors in stimulating plant development^23^. Recently, understanding how rhizobacteria build biofilms in association with plant surfaces has been the subject of numerous investigations^24–26^. In soil ecosystems, interactions between bacterial populations and plant roots primarily occur in the biofilm mode rather than the planktonic mode of existence. These interactions can result in either positive or negative outcomes ^27,28^.

Our investigation was built on three experiments, including *in vitro* biofilm-forming and PGP traits and *in vivo* evaluation of biofilm-forming rhizobacteria for wheat productivity. For this purpose, ten most robust isolates were selected with biofilm forming ability (Fig. 1A). It has been found that one crucial strategy for the bacterial strains' efficient survival in the plant rhizosphere is their capacity to create biofilms. An earlier study found a substantial positive association between root colonization and *in vitro* biofilm development. Therefore, these PGPR should be effective at avoiding competing species, absorbing nutrients, and responding quickly to changing environmental conditions by creating biofilms. The capacities of the plants' associated biofilms allow them to protect themselves against external pressures and other microbial competition in the rhizosphere, as well as to provide favorable effects that promote plant growth ^29^. The potential to produce EPS of these isolates were also determined as shown in Fig. 1B. It is established that biofilms are communities of microbial cells attached to surfaces and enclosed within a self-produced extracellular polymer shell (EPS). This shell predominantly consists of proteins, polysaccharides, extracellular DNA, and lipids^30^. The polysaccharide band region is primarily caused by the stretching vibration of C–C and C–O bonds, as well as the deformation of C–O–H and C–O–C bonds, wherein negatively functional groups bind with heavy metals to remove them from environment ^31,32^. The colonization of bacterial roots by diverse functional groups is also crucial ^33^. It has been found most of these isolates were *Bacillus* sp. according to their morphological characteristics. According to reports, PGPR, particularly *Bacillus*, effectively promotes plant growth ^34^. While two bacterial strains BF15 and BF10 with promising PGP traits were identified as *Lysinibacillus macrolides* SB001 (ON725075) and *Brucella* sp. SB002 (ON705758) based on 16s rRNA partial gene sequencing as illustrated in Fig. 2.

In the current study the biofilm producing PGPR have shown the potential to produce IAA from 6.8 to 15.4 µg mL^−1^ and most of the biofilm producing rhizobacteria have capacity to produce siderophores, phosphorus and potassium solubilization and tolerance to salinity as mentioned in Table 1. The phytohormones produced by PGPR play a direct role in promoting plant growth. Among these phytohormones, IAA increases the number of lateral roots, root hairs, and this increased root surface area helps the plants absorb water and nutrients even during droughts ^35,36^. Therefore, for healthy plant growth and development, a constitutive synthesis of IAA is necessary. Due to its insoluble nature, phosphorus is unavailable for plant use ^37^. *Bacillus, Enterobacter, Gluconacetobacter, Pseudomonas,* and *Serratia* are among the genera of PGPR that are known to play a crucial role in solubilizing insoluble forms of phosphorus. This is primarily due to their ability to produce acid phosphatases, which aid in the mineralization of organic phosphorus in soil ^38,39^. Similarly, PGPR synthesize organic acids such as citric and gluconic acid, which facilitate the solubilization of phosphorus. This process enhances plant growth and productivity by making these mineralized or soluble molecules available for absorption ^37^. In the present study, biofilm forming PGPR are positive for IAA with variable degree of efficacy, while 7 out of 10 strains showed PS activity. The *in vitro* and *in vivo* growth promotion of wheat plants upon inoculation might be due to their IAA and PS activities as shown in Table 1.

Additionally, generation of organic and inorganic acids, including citrate, oxalate, acetate, sulfuric acid, carbonic acid, and nitric acid, promotes the solubilization of other elements, like potassium and zinc, which are crucial for improving crop yield and soil fertility^38^. Iron is a naturally occurring element that is acquired through the production of Fe-siderophore complexes. Siderophores are iron-chelating substances that bacteria release. They help plants and endophytes consume iron by reducing it from Fe^3+^ to Fe^2+^ both intra- and intercellularly. When there is a lack of iron in the environment, siderophores play an important role^40^.

Several examples of biofilm PGPR that are significantly more effective in field settings than any planktonic PGPR. According to Backer et al. ^41^ biofilm PGPR exhibits much higher levels of nitrogenase activity, ammonia synthesis, phosphate solubilization, IAA formation, and siderophore production in comparison to planktonic PGPR. Biofilm forming PGPR also confer advantages such as enhanced resistance to antibiotics and survival in unfavorable environmental conditions such high temperatures, severe pH, salinity, and drought. These factors raise the organism's probability of thriving in a competitive soil environment^42^.

Surface-enhanced Raman spectroscopy (SERS) is an effective tool for identifying biofilm-forming bacterial strains and the components associated with biofilm ^43,44^. The biofilm components i.e., carbohydrates, polysaccharides, protein, and lipids are involved in surface adhesion of biofilm-forming PGPR on plant tissues ^45^. The peak intensity of SERS spectra explained the relative efficacy of biofilm production by selected bacterial strains. The data in (Fig. 3) further depicted the strains BF10 and BF15 are more efficient in biofilm production relative to other strains.

*Bacillus* species have garnered significant attention among PGPR, due to their remarkable ability to form biofilms ^46,47^. The current study found that the PGPR inoculated plants performed significantly better than the untreated control in terms of seed germination, and root-shoot length (Table 3, 4). Previous study results showed that PGPR may improve seed germination by decreasing the prevalence of seed mycoflora, which can be harmful to plant health ^48^. Study data of Duarah et al. ^49^ suggested that inoculating rice and legume seeds with PGPR increased amylase activity during germination. Moreover, amylase transforms starch into metabolizable sugars that sprouting seedlings use to grow their roots and branches ^50^. One of the processes of PGPR that is most frequently cited is the production of phytohormones like indole-3-acetic acid-IAA ^51^. It is generally known that the auxin IAA has a significant influence on root architecture and growth. Exogenous IAA of rhizobacterial origin can boost plant development by enhancing the length and generating more biomass from roots by modulating the expression of host genes involved in auxin response, defense, hormones, and cell wall formation ^36,41,52^. Similar studies by other researchers have revealed that PGPR often produces IAA ^53,54^. IAA is essential for promoting plant growth since it increases root formation and nutrient uptake ^55^. In our investigations, it was found that the bacterial inoculation that produces biofilm improved plant dry mass, number of tillers, and grain yield (Figs. 4, 5 and Table 4). Phytohormones substantially impact plant growth and development, as well as act as a player to cope biotic and abiotic stress ^56^.

Gibberellin, cytokinin, and IAA production correlate with the PGPR, which promotes crop productivity ^57,58^. It has been reported that these hormones are also formed by several *Lysinibacillus* species ^59,60^. According to various scientific research groups, *Lysinibacillus* inoculation significantly improved crop growth and production ^61,62^. According to Zhou et al. ^63^, the PGPR needs significant root colonization in order to successfully establish in the rhizoplane and rhizosphere to enhance crop growth. Root colonization by PGPR is typically stimulated by the production of biofilms on root surfaces that aid in moisture retention and protect plant roots from harmful microorganisms^64^. Several studies have shown that beneficial bacteria adhere to plant roots and are crucial for nutrient cycling, phytopathogen management, and ultimately higher crop output. These plants include rice, wheat, maize, cucumber, and a variety of legumes^65,66^. However, PGPR are effective in colonizing plant roots, and after a successful plant-microbe interaction, they can grow into microcolonies or produce biofilm. These plant-associated biofilms are extremely capable of protecting against external stress, lowering microbial competition, and offering the host plant favorable conditions that boost growth, yield, and crop quality^67,68^. The improved solubilization and availability of the nutrient supply, as well as greater osmo-protection, might be the potential contributor to increase in production^68,69^. Similarly, our study findings depicted that inoculation has a significant positive response on chlorophyll, soil moisture, and nutrient contents (Figs. 6, 7).

The inoculated PGPR accelerated nitrogen fixation may be the reason for the increased pigment synthesis. Nevertheless, nitrogen has a key role in chlorophyll's structural makeup. In the case of soybean plants, *Bacillus pumilus* increases the production of soluble proteins^70^. Because PGPR-mediated phytohormones play a key role in integrated nutrient management, root proliferation, and exopolysaccharide synthesis, thereby positive effects on plant growth and yield^3,71^. This may explain why soil moisture and nutrient levels increased in our study. By modifying the root morphology, these PGPR increase root surface area for nutrient uptake from soil and protect crops against different diseases^72^.

EPS are yet another PGPR byproduct that helps in biofilm production and retention^73^. EPS act as the best matrix for holding soil moisture and adhering to soil particles, as a result preventing the roots from drying out^45,74^. The higher mineral content may be responsible for the higher root biomass, growth and development resulting in the positive effects on microbial activity. In another study, it was shown that EPS produced through biofilms increased permeability by aggregating the soil and preserving a higher water potential near the root zone. This improved nitrogen uptake by plants and protected them from water shortages^75^. Therefore, these results are consistent with our findings that inoculation with promising biofilm-forming bacterial strains (isolates from the wheat rhizosphere) improves soil quality by increasing microbial activity in the rhizosphere, enhancing nutrient content, plant yield, and soil quality. This suggests that such strains can act as alternative agents in integrated nutrient management. Recent studies have demonstrated that *Lysinibacillus* species, such as *L. sphaericus*, *L. fusiformis*, *L. chungkukjangi*, and *L. xylanilyticus*, also have a number of beneficial traits related to improving plant growth and development.

Moreover, it has been reprted that the most limiting element for plant growth and productivity is nitrogen andplants absorb nitrogen from the soil through their roots, but the rhizosphere has a limited amount of accessible nitrogen. Although nitrogen gas makes up over 80% of the earth's atmosphere, plants cannot use it in such form^76^. A few different species of *Lysinibacillus* have been shown to have the ability to turn nitrogen into ammonia^77–79^. Bacteria fix nitrogen through the catalytic activity of a complex enzymatic system called nitrogenase, which is encoded by Nif genes. Studies revealed that nitrogenases are produced by and Nif genes are present in *Lysinibacillus* species^80^. Among the three essential macronutrients, phosphorus (P) is needed in several different metabolic pathways necessary for plant growth. Numerous *Lysinibacillus* species have been found to have the ability to convert fixed inorganic P compounds into soluble P forms that can be easily taken up by plants. Additionally, a number of *Lysinibacillus* strains have the ability to dissolve other important insoluble minerals, including potassium, iron, zinc, and silicate^81,82^. The conversion of insoluble minerals into bioavailable forms has been found to be facilitated by the release of organic acids, hydrolytic enzymes, and metal chelator compounds from certain *Lysinibacillus* spp.^83,84^.

Further, Faisal and Hasnain^85^ investigated that the *Bacillus cereus,* and *Brevibacterium* colonized the rhizoplane and root zone of *Triticum aestivum* plants, and this colonization was favorable to both the bacteria and the plants. Furthermore, it was demonstrated that *Ochrobactrum intermedium* and *Bacillus cereus* enhanced the growth, root and shoot length, weight and quantity of seeds per pod, and number and value of grains per plant of *Lens esculenta*. Taken together, it is suggested that *Ochrobactrum* isolates could improve plant growth by promoting nutrient uptake, participating in symbiotic nitrogen fixation, and preventing plant diseases in *Acacia mangium* ^86^, tea^87^, cucumber^88^, and mung bean^89^.

Thus, the PGPR strains had variable effects for improving the growth, physiology, yield and nutrient uptake of wheat under controlled and natural soil conditions. Interestingly, the inoculation with selected PGPR strains showed promising results under pot and field conditions but with variable efficacy when compared with uninoculated control. The superiority of the strains BF15 and BF10 may also be related to the presence of other characteristics such as indole acetic acid production, phosphorus/potassium solubilization, and siderophore production in addition to their biofilm-forming ability, which made them more effective or competitive under natural field conditions.

## Conclusion

In agricultural systems, the incorporation of few PGPR have been challenging despite extensive research, primarily due to their low survival rates across various soil conditions. The study demonstrates that biofilm-forming plant growth-promoting rhizobacteria (PGPR) have significant potential to enhance crop yields and improve agricultural sustainability. By isolating and characterizing native rhizobacteria from the wheat rhizosphere, the research identified promising strains capable of forming biofilms and promoting plant growth through various traits. Under controlled and field conditions, these biofilm-producing strains consistently showed beneficial effects on wheat growth and yield attributes, including larger roots and shoots, increased biomass, plant height, grain yield, tiller numbers, and nutrient content (nitrogen and phosphorus). Surface-enhanced Raman Spectroscopy (SERS) analysis further provided insights into the biochemical composition of the biofilms produced by these strains, highlighting their complex structure composed of proteins, carbohydrates, lipids, amino acids, and nucleic acids. The identification of *Brucella* sp. (BF10) and *Lysinibacillus macroides* (BF15) as the most promising biofilm-producing isolates underscores their potential for application in sustainable agriculture practices. Overall, the findings support the conclusion that biofilm-forming PGPR can effectively enhance crop productivity by improving growth and physiological responses, thereby contributing to sustainable agricultural practices.

## Materials and methods

### Isolation of rhizobacteria

Soil samples from wheat rhizosphere were aseptically collected from fields at Ayub Agricultural Research Institute, Faisalabad, Pakistan, and stored in sterile aluminum foil at 4°C until isolation of plant growth-promoting rhizobacteria. Fifty-six rhizobacterial isolates were isolated from collected rhizosphere soil (1 g) adhering to roots by serially diluting up to 10^−7^ (supplementary file 1). Nutrient agar plates were inoculated with the serially diluted soil solution and incubated at 28 ± 2 °C for 48 h. Morphologically different individual colonies were taken and re-streaked several times on nutrient agar plates for purification. Then purified bacterial isolates were preserved in 50% glycerol stock at − 20 °C and in nutrient agar slants at 4 °C for further characterization studies.

### Screening for biofilm formation and exopolysaccharide production

*In vitro* biofilm formation by the rhizobacterial isolates was examined using the method of O’Toole and Kolter^90^. Bacterial isolates were initially grown in a nutrient broth overnight at 100 rpm agitation, and optical density was recorded at 600 nm (OD_600_). The cultures were centrifuged at 12000 rpm for 10 min, and the supernatant was discarded. Afterwards the pellet was resuspended in sterile distilled water and then diluted up to 10^5^ colony-forming units (CFU) mL^−1^. Further, 50 μL cultures were inoculated in 96-well polystyrene microtiter plates containing nutrient broth medium and incubated at 28 ± 2 °C for 48 and 96 h under static conditions. Formation of pellicles was observed, and isolates were termed biofilm-forming bacterial strains. Biofilm formation was quantified through staining techniques. The microtiter plates were emptied after incubation and gently washed with sterile distilled water to remove any loosely attached bacterial cells and microtiter plate wells were stained with 0.1% (*w/v*) crystal violet solution (200 µL) following incubation for 45 minutes. Each microtiter plate well was rinsed three times with sterile distilled water, and crystal violet was eluted using 95% ethanol. Biofilm formation was quantified by measuring the optical density at 595 nm (OD_595_) using an ELISA plate reader (Thermo Scientific Multiskan EX, UK). The exopolysaccharide (EPS), production was quantified by following the standard method of Mody et al.^91^. Nutrient broth (50 mL) was inoculated with 500 μL of freshly grown bacterial culture and kept at 28 ± 2 °C for five days on 100 rpm agitation. Cultures were centrifuged at 10,000×g for 10 minutes at 4 °C and filtered through a 0.45 μm nitrocellulose filter. Chilled ethanol was added to the culture filtrate (1:1 ethanol: liquid culture ratio) to precipitate EPS. Further precipitated EPS was dried at 80 °C for 48 h and weighed to determine EPS production by bacterial strains.

### Morphological and biochemical characteristics

Ten biofilm-forming rhizobacterial strains (BF10, BF15, BF18, BF20, BF22, BF24, BF27, BF28, BF32, and BF37) were characterized for morphological and biochemical traits. A compound microscope (100X) was used for morphological characterization to observe colony shape and cell shapes, size, motility, and aerobic nature. The Gram-reaction of target rhizobacterial strains was performed by preparing a microscopic slide stained with crystal violet, iodine solution, and safranin, as reported by Mudili^92^. Biochemical tests, including catalase, oxidase, glucose utilization, and starch hydrolysis, were performed using the standard protocols of Cappuccino and Sherman^93^.

### Determination of in vitro PGP characteristics

All the ten biofilm-forming rhizobacterial strains were also characterized for PGP traits, *viz*., production of indole acetic acid (IAA), siderophores, phosphate (P), potassium (K) solubilization, and salt-stress tolerance. IAA production by bacterial strains was determined calorimetrically in the presence of L-tryptophan^94^. Bacterial cells were cultured at 28 ± 2 °C for 72 h and 120 rpm in tryptic soy broth enriched with 1% L-tryptophan. After 72 h of incubation, the bacterial cells were extracted by centrifugation (10,000 g for 10 min), and culture supernatants (3 mL) were mixed with 2 ml of Salkowski's reagent (2 mL FeCl_3_ (0.5 M) and 98 mL perchloric acid (35%). After 30 minutes of incubation, the solution was read at 535 nm with a spectrophotometer, and IAA concentration was quantified by plotting standard curves. The siderophore production by bacterial strains was assessed through spot inoculation on chrome azurol S (CAS) blue agar plates and incubated at 28 ± 2 °C for 7 days^95^. Formation of orange halo zones around the colony growth was observed for siderophore production by bacterial strains.

P-solubilization by bacterial strains was determined using Pikovskya agar medium containing tri-calcium phosphate as an insoluble P source. While K-solubilization by bacterial strains was determined using an Aleksandrov agar medium containing mica as an insoluble K source. Freshly grown bacterial cells were spot inoculated on Pikovskya and Aleksandrov agar media and incubated at 28 ± 1 °C for five days. Appearance of a halo zone around the bacterial colonies was reported as positive results for P and K-solubilization by bacterial strains. Salt tolerance of biofilm-forming bacterial strains was evaluated by streaking bacterial colonies on a nutrient agar medium containing 0.5–2.5% (*w/v*) NaCl^96^. The growth of bacterial strains at the respective salt stress levels was considered salt-stress tolerant.

### Culturing biofilm-forming rhizobacteria for acquisition of surface-enhanced Raman spectroscopy (SERS) spectra

Based on efficient PGP traits, the selected five rhizobacterial isolates were further tested for biofilm formation through surface-enhanced Raman spectroscopy (SERS). Selected bacterial strains BF10, BF15, BF20, BF24 and BF27 were cultured in 24 wells microtiter plate containing nutrient broth for 48 hours in an incubator at 28 ◦C ± 2. For the SERS spectral acquisition from bacterial biofilm, silver nanoparticles were prepared by using chemical reduction method to be used as SERS substrate. Before acquiring spectra, the bacterial biofilm was washed with saline buffer solution. The EPS layer of each biofilm forming bacterial isolates was separated by spatula and mixed with 50 µL of silver NPs in Eppendorf tube and left for half an hour for incubation time. The spectral acquisitions were performed using a Raman Spectroscopy, Peak Seeker Pro-Agiltron, (USA) equipped with a 785 nm laser as source delivering 50 mW laser through a 10X objective with an integration time of 15 s.

### Molecular identification of selected biofilm-forming rhizobacteria

Two most promising biofilm-forming rhizobacterial strains, BF10 and BF15, were recoded with SB002, SB001 and identified through 16S rRNA partial gene sequencing using the commercial service of Macrogen, Seoul, Korea. Universal primers 27F and 492R were used to amplify the 16S RNA gene by polymer chain reaction (PCR). Gene sequences were compared with existing sequences in the GenBank database using the blast tool on the NCBI server and gene sequences with more than 98% identity were retrieved and aligned using the Clustal-W method. A neighbor-joining phylogenetic tree was generated using Mega software version 11.0.13^97^ with 10,000 bootstrap values. Furthermore, more than 50% of bootstrap values were shown in the tree. The identified bacterial strains SB001 and SB002 (BF15, BF10 respectively) sequences were deposited to GenBank, NCBI, under the accession numbers ON725075.1 and ON705758.1, respectively.

### Screening for plant growth promotion under axenic conditions

A growth chamber experiment was also carried out to assess the impact of ten biofilm-forming rhizobacterial strains (BF10, BF15, BF18, BF20, BF22, BF24, BF27, BF28, BF32, and BF37) on wheat seed germination and growth attributes. Bacterial inoculum was grown in nutrients broth at 28 ± 2°C for 48 h in a shaking incubator. The optical density (OD_595_) of the inoculum was adjusted to 0.5 measured by using spectrophotometer (Evolution 300 LC, Cambridge, UK) to obtain a uniform population of bacteria (10^8^ –10^9^ CFU mL^–1^) in the broth at the time of inoculation. The wheat seeds were surface sterilized with 3% sodium hypochlorite for 2 minutes and carefully rinsed in sterile distilled water. The 100 µL of inoculum was added to some volume of the peat and then coated on wheat seeds in 1:1 ratio. Seeds soaked in sterilized distilled water were used as the control. Afterwards, seeds were placed over the sterile filter paper in a petri dish and covered with a tight-fitting lid. Then the petri plates were kept in an incubator maintaining the moisture and temperature of 28 ± 2 °C. The seed germination assay was performed through a completely randomized design (CRD) layout with three replications. Germinated seeds were counted after four days after planting (DAP), and the germination percentage was calculated. The experiment was harvested after two weeks of planting, and root, shoot length and mass were recorded.

### Pot and field trials

Pot and field scaled trials were carried out in the wire house and farm area of the Soil Bacteriology Section, Ayub Agriculture Research Institute, Faisalabad, Pakistan, to evaluate the effect of biofilm-forming rhizobacterial strains on the wheat growth and yield. The experiments were conducted in the area located at latitude 31.4187 ^o^N, longitude 73.0791 ^o^E, and elevation 186.0 m above sea level. The average minimum and maximum temperatures during wheat crop season were 9.2 °C and 23.6 °C, respectively. The experimental location has a subtropical climate and receives 25 mm of rainfall annually. The soil was analyzed for pH^98^, organic matter^99^, total N, available P, and extractable K by following^100^. The texture of the soil was sandy clay loam in both pot and field experiments with 7.79 pH, 0.78% organic matter, 0.033% total nitrogen, 7.69 mg kg^−1^ available phosphorus 121 mg kg^−1^ extractable potassium. For the pot experiment, the soil was air-dried, sieved (2 mm), and used to fill pots (16 kg capacity).

Based on their PGP traits and growth chamber study, top five promising biofilm-forming rhizobacterial strains (BF10, BF15, BF20, BF24, and BF27) were chosen for pot and field trials. These strains were freshly cultured on nutrient agar to coat wheat seed. Surface disinfected wheat seeds (variety Akbar 2019) were inoculated with reference culture mixed with peat (sterilized) and 25 mL raw sugar solution (10%). The seeds were treated with an uninoculated broth culture mixed with peat and 25 mL raw sugar solution for control (10%). Six inoculated wheat seeds (provided by Agronomy department, AARI) were sown in each pot, and pots were arranged in completely randomized design (CRD) arrangements having three replications. After seed germination, four plants were maintained in each pot. Field study was conducted for two consecutive years 2020–2021 and 2021–2022. Coated seeds were sown at 80 kg ha^−1^ in a plot size of 15.0 m^2^ (2.5 m × 6.0 m). The treatments were sown in randomized complete block design (RCBD) arrangements in triplicates. The recommended dose of chemical fertilizer 120-80-60 NPK kg ha^−1^ in the form of Urea, Single super phosphate (SSP), and Muriate of potash (MOP), respectively, were applied in both pots and field experiments. Half N and total doses of P and K were mixed in soil at the sowing time, and the remaining N was applied with the first two irrigations in equal splits. Both pot and field trials were maintained up to physiological maturity, and data regarding growth, biochemical, physiological, and nutrient acquisition were observed.

### Morphological and physiological observations

The germination rate was estimated after 96 h of seed sowing by calculating the percentage of total number of normal seedlings to the seeds planted. Several growth and yield attributes, e.g., plant height, fresh and dried shoots, root weight, root length, grain yield, tiller count, spike length, and 1000 grain weight, were recorded when harvesting. For the measurement of plant height under field conditions, from each replication of treatment, five randomly plants were selected, while yield data of m^2^ was recorded avoiding borders. After 60 days of sowing, one gram of wheat plant leaf sample was collected in pre-weighed, clean glass vials with 10 mL of acetone (80%) to test the chlorophyll content. Leaf material was bleached before being drained out. A spectrophotometer (Spectronic Genesys-5, Milton Roy) was used to read the optical density at 663 and 645 nm using acetone (80%) as a blank. The concentration of chlorophyll a and chlorophyll b (μg g^–1^) was calculated according to Lichtenthaler and Wellburn^101^. Relative water content (RWC) of leaves was estimated by recording fresh leaf weight. Then the leaves were soaked in distilled water for 24 h to record the turgid leaves weight. The constant oven-dried weight was taken after drying the leaves in the oven at 70 °C, and RWC was calculated as reported by Davenport^102^. Following formula was used to determine relative water contents:

$${\text{Relative }}\,{\text{water }}\,{\text{contents }}\left( \% \right) = \left[ {\left( {{\text{Fresh }}\,{\text{mass}} - {\text{dry }}\,{\text{mass}}} \right)/{\text{Saturated}}\,{\text{ mass}} - {\text{dry }}\,{\text{mass}}} \right)]*{1}00$$

From the effective root zone of the wheat plant's which is 0 to 90 cm-long, soil samples were collected to determine soil moisture according to Hou et al.^103^ and formula is given below:

$${\text{Soil }}\,{\text{moisture}}\,{\text{ contents }}\left( \% \right) = \left( {{\text{Fresh}}\,{\text{ soil }}\,{\text{weight}} - {\text{ Oven }}\,{\text{dry }}\,{\text{weight}}/{\text{Oven}}\,{\text{ dry}}\,{\text{ weight}}} \right)*{1}00$$

After harvest, plant straw and grain samples were grounded after oven-drying at 67 °C and 0.1g of each sample was wet-digested as reported by Wolf^104^. The N concentration was determined using the Kjeldahl method, while the P concentration was determined using the colorimetric method^105^.

### Statistical analysis

The analysis of variance (ANOVA) was performed on collected data related to plant growth, yield, and biochemical parameters. The least significant test (LSD) was accomplished through the statistical software SPSS to determine significant differences among treatment means^106^. The correlation matrix and principal component analysis were performed using R software version 4.1.2.

The raw SERS spectral data of samples was pre-processed for removal of noise and baseline correction to get useful information. MATLAB 7.8 version was used to perform the pre-processing of SERS raw data by employing chemometric codes^107^. Firstly, all SERS raw data was imported in MATLAB in a single matrix and then pre-processed by algorithms for baseline correction, smoothening, substrate removal and vector normalization. Savitzky–Golay is an algorithm that was applied for smoothening purposes while polynomial methods and rubber band correction methods were used for the baseline correction.

### Ethical approval

The study was in accordance with relevant institutional, national, and international guidelines and legislation.

### Supplementary Information

Supplementary Information.

## Data Availability

The original data presented in the current study can be made available on a reasonable request from corresponding author.
